# Editorial: Reviews in bacteria and host

**DOI:** 10.3389/fcimb.2026.1961702

**Published:** 2026-09-03

**Authors:** Percy Schröttner, Hani Harb

**Affiliations:** 1 Institute for Medical Microbiology and Virology, Faculty of Medicine and University Hospital Carl Gustav Carus, Technische Universität Dresden (TUD) Dresden University of Technology, Fetscherstraße 74, Dresden, Germany; 2 Institute for Clinical Chemistry and Laboratory Medicine, Faculty of Medicine and University Hospital Carl Gustav Carus, Technische Universität Dresden (TUD) Dresden University of Technology, Fetscherstraße 74, Dresden, Germany

**Keywords:** infectious diseases, microbiome, organoids, pathogen-host interaction, review

The outcome of bacterial infection is not determined by microbial virulence or host immunity in isolation, but by a continuously evolving interaction across molecular, cellular, tissue, organismal, and ecological scales. Bacteria sense and remodel host environments, exploit anatomical niches, interfere with intracellular trafficking and metabolism, and adapt to immune pressure. Hosts, in turn, deploy layered physical barriers, innate and adaptive immune responses, fever, nutritional restriction, and tissue-specific repair programs. The Research Topic “Reviews in Bacteria and Host” brings together 16 reviews and mini-reviews that capture this complexity while emphasizing a central translational question: how can mechanistic understanding of host–pathogen interactions be converted into better experimental models, biomarkers, preventive strategies, and therapies? Collectively, the contributions span veterinary and human infections, acute and persistent disease, canonical pathogens and commensal opportunists, the microbiome, cancer, and emerging model systems. The manuscripts were divided into three main themes.

The first group of articles examined the experimental platforms through which host–pathogen biology is studied. Weldearegay et al. review primary culture systems for bacterial respiratory infections of livestock, including nasal mucosa explants, tracheal organ cultures, air–liquid interface cultures, and precision-cut lung slices. These models preserve tissue architecture and differentiated epithelial functions more effectively than conventional immortalized monolayers, while also supporting the 3R principle through the use of abattoir-derived material. Their discussion makes clear, however, that model fidelity must be balanced against limited culture duration, donor variability, restricted immune-cell recruitment, and technical complexity. Shaw et al. extend this methodological perspective to stem-cell and organoid systems for investigating host–microbe interactions in low-biomass tissues. Organoids can reconstruct organ-specific cellular organization and permit genetic or mechanistic interrogation, but contamination control, incomplete immune and vascular compartments, and the need for careful validation remain decisive concerns. Together, these reviews position advanced ex vivo systems not as universal replacements for animal models, but as complementary tools within an integrated experimental pipeline.

Two further contributions illustrate how reductionist and alternative models can be selected according to the biological question. Primm et al. assess *Galleria mellonella* larvae as a tractable model of *Campylobacter jejuni* infection. The system is inexpensive and informative for comparative virulence, innate immune responses, capsule function, transcriptional regulation, outer-membrane vesicles, and temperature-dependent phenotypes. At the same time, high-dose hemocoel injection does not reproduce the complex, anaerobic, microbiota-rich human intestinal environment, highlighting the importance of refining oral infection approaches. Garomsa et al. provide a broader comparative analysis of *in vitro* and *in vivo* models for *Acinetobacter baumannii*, a pathogen whose multidrug resistance intensifies the need for new antimicrobials, vaccines, and immunotherapies. Their review emphasizes that cell lines, *G. mellonella*, and murine systems answer different questions and that strain heterogeneity, inoculum, multiplicity of infection, incubation time, tissue relevance, and immune competence must be standardized. Across these papers, model selection emerges as a hypothesis-driven decision rather than a matter of convenience.

A second major theme is microbial persistence through subversion of intracellular defense and exploitation of protected tissue niches. Li et al. synthesizes the dual role of autophagy in mycobacterial infection. Autophagic delivery to lysosomes is an important host-defense mechanism, yet mycobacteria can inhibit, redirect, or exploit this machinery to support intracellular survival. This tension provides a rationale for host-directed therapies that activate protective autophagy, including in drug-resistant infection, while demanding careful assessment of context-dependent effects. Sundaram and Prabhu examine progression from latent *Mycobacterium tuberculosis* infection to active disease and connect bacterial genes, impaired autophagosome–lysosome fusion, macrophage regulation, non-coding RNAs, and T-cell alterations to loss of immune containment. Their synthesis underscores the need to combine microbial determinants with host biomarkers rather than searching for a single predictor of reactivation. Du et al. similarly describe how *Nocardia* species invade cells, resist oxidative stress, acquire iron, damage membranes, and persist within tissues through factors such as Mce proteins, antioxidant enzymes, phospholipase C, hemolysins, and siderophore-associated proteins. The review also highlights neutrophils and the γδ T-cell–IL-17 axis as key components of protection, while noting that mechanistic knowledge remains concentrated in a limited number of reference strains.

Persistence can also be spatially organized. Rong et al. focus on *Staphylococcus aureus* invasion of the osteocyte lacuna–canalicular network in chronic osteomyelitis. This mineralized microanatomical compartment provides a physical sanctuary that complements better-known persistence mechanisms such as biofilms, intracellular residence, and abscess formation. The concept helps explain why bone infection may relapse despite prolonged antimicrobial therapy and surgical intervention, and it identifies the need for therapies capable of reaching and eliminating bacteria within the canalicular network. Branford et al. address a related but distinct staphylococcal problem at the interface of commensalism and disease. Their review of *Staphylococcus pseudintermedius* and canine immunity integrates adhesins, exoenzymes, cytotoxins, biofilm formation, complement inhibition, degradation of neutrophil extracellular traps, cytokine modulation, genomic adaptation, and antimicrobial resistance. Host keratinocyte, neutrophil, and Th1/Th17 responses may promote clearance but also contribute to inflammatory tissue injury. This One Health perspective is particularly valuable because it links veterinary disease, methicillin resistance, immune evasion, and vaccine-antigen discovery.

The Research Topic also revisits host responses that can be simultaneously protective and pathogenic. Kramarczyk et al. conceptualize infectious fever as a cycle initiated by microbial pyrogens and host inflammation, followed by thermal effects on both immune function and bacterial physiology. Febrile temperatures can disrupt bacterial membranes, redox balance, gene regulation, metabolism, and replication, but pathogens may also mount adaptive stress responses. By considering the consequences of commonly used antipyretics, the review challenges an overly simplistic view of fever as merely a symptom to suppress and calls for pathogen-, host-, and context-specific evaluation. Mathew et al. explore a comparable duality in Group A Streptococcus infection. Although vaccine research has largely pursued antibody correlates, GAS-specific T cells may contribute substantially to protection. Th17 responses may support mucosal defense, whereas superantigen-driven activation and pathogenic CD8+ T-cell responses can promote immunopathology. Defining the phenotype, specificity, anatomical localization, and timing of these T-cell responses may therefore be crucial for rational vaccine design and for avoiding immune-mediated complications.

A third cluster of reviews expands the host–pathogen framework to microbial communities and their metabolites. Zhao et al. examine the establishment of gut and lung microbiota across early life, including the controversy surrounding microbial detection *in utero* and the special analytical challenges of low-biomass samples. After birth, delivery mode, feeding, antibiotics, and environmental exposures shape microbial succession, while bidirectional gut–lung communication influences immune and metabolic maturation. Early dysbiosis is consequently linked to allergic, respiratory, and metabolic disease, yet microbiome-based interventions require rigorous evaluation of causality, safety, developmental timing, and ethics. Bu et al. review gut microbial alterations and microbial metabolism in autism spectrum disorder. They discuss reduced diversity, altered community structure, metabolites such as short-chain fatty acids, phenolic compounds, bile acids, and amino acids, and their potential effects on barrier integrity, neuroinflammation, neurotransmission, and the microbiota–gut–brain axis. The therapeutic promise of dietary, probiotic, prebiotic, and microbiota-directed approaches is balanced by heterogeneity among cohorts and the difficulty of distinguishing causal mechanisms from disease-associated signatures.

Microbial influences on carcinogenesis are addressed from complementary perspectives. He et al. delineate how *Helicobacter pylori* modifies host signaling, inflammation, DNA-damage responses, cell junctions, genomic stability, and proliferative programs during gastric carcinogenesis. Their review demonstrates that cancer-promoting infection is not reducible to a single virulence factor or pathway; rather, it results from sustained crosstalk among bacterial effectors, host signaling networks, chronic inflammation, and tissue damage. He et al. extend this systems view to the microbiota–host metabolic axis in cervical cancer. Persistent high-risk human papillomavirus infection remains the prerequisite for cervical carcinogenesis, but vaginal dysbiosis and co-metabolic remodeling may facilitate viral persistence, epithelial barrier disruption, local immune evasion, oncogenic signaling, and therapy resistance. This contribution moves beyond taxonomic description toward functional metabolites and metabolic networks, thereby identifying microbiota-targeted strategies as potential adjuncts to cancer prevention and treatment while emphasizing the need for mechanistic and clinical validation.

Finally, Dădârlat-Pop et al. translate host–microbe complexity into a rapidly evolving clinical setting: infective endocarditis following transcatheter aortic valve implantation. TAVI-associated endocarditis differs from surgical prosthetic-valve endocarditis in patient frailty, microbiological profile, imaging performance, and therapeutic feasibility. The prominent contribution of enterococci, the limitations of echocardiography, and the added value of cardiac computed tomography and 18F-FDG PET/CT illustrate why diagnosis requires multimodal assessment. Because operative risk, comorbidity, pathogen, prosthetic complications, and likelihood of source control vary substantially, management must be individualized by an experienced Heart Team. Prevention, including procedural infection control and attention to healthcare-associated exposure, is therefore as important as treatment.

Taken together, these 16 contributions show that the next phase of host–pathogen research will depend on integration across scales. Mechanistic studies must be anchored in models that preserve relevant tissue architecture, immune context, microbial diversity, and temporal dynamics. Omics and single-cell technologies can reveal candidate pathways and biomarkers, but spatial validation, functional perturbation, reproducibility, and clinically representative cohorts are essential. Likewise, host-directed, microbiome-directed, vaccine, and metabolic interventions will require stratification according to pathogen, anatomical niche, disease stage, host background, and treatment history. The Research Topic also reinforces the value of One Health thinking: insights from livestock, companion animals, alternative infection models, and human disease are mutually informative. By bringing experimental methodology, bacterial adaptation, immune regulation, microbial ecology, and clinical decision-making into a common framework, this Research Topic provides both a synthesis of current knowledge and a roadmap for more predictive, mechanistically grounded, and translationally useful studies of bacteria and their hosts.

